# Smart Sensor Architectures for Multimedia Sensing in IoMT

**DOI:** 10.3390/s20051400

**Published:** 2020-03-04

**Authors:** Javier Silvestre-Blanes, Víctor Sempere-Payá, Teresa Albero-Albero

**Affiliations:** 1 ITI and Universitat Politècnica de València (UPV), DISCA, EPSA, 03801 Alcoy, Spain; talbero@disca.upv.es; 2 ITI and Universitat Politècnica de València (UPV), DCOM, ETSIT, 46022 Valencia, Spain; vsempere@dcom.upv.es

**Keywords:** IoMT, governor, edge computing, near sensor computing

## Abstract

Today, a wide range of developments and paradigms require the use of embedded systems characterized by restrictions on their computing capacity, consumption, cost, and network connection. The evolution of the Internet of Things (IoT) towards Industrial IoT (IIoT) or the Internet of Multimedia Things (IoMT), its impact within the 4.0 industry, the evolution of cloud computing towards edge or fog computing, also called near-sensor computing, or the increase in the use of embedded vision, are current examples of this trend. One of the most common methods of reducing energy consumption is the use of processor frequency scaling, based on a particular policy. The algorithms to define this policy are intended to obtain good responses to the workloads that occur in smarthphones. There has been no study that allows a correct definition of these algorithms for workloads such as those expected in the above scenarios. This paper presents a method to determine the operating parameters of the dynamic governor algorithm called *Interactive*, which offers significant improvements in power consumption, without reducing the performance of the application. These improvements depend on the load that the system has to support, so the results are evaluated against three different loads, from higher to lower, showing improvements ranging from 62% to 26%.

## 1. Introduction and Related Work

The development of the IIoT (Industrial Internet of Things) and ICPS (Industrial Cyber Physical Systems) paradigms [1] introduced by the Industry 4.0 concept [2] is producing an increase in the use of embedded systems. This in turn is creating new needs in industry [3]. The use of more distributed devices in plants, which collaborate to achieve a certain goal, is one of these needs. This trend has created new communications requirements, as well as requirements in terms of their computational capacity and energy consumption. Moreover, there is a trend in the use of the Internet of Things (IoT) towards mobile, multisensorial and smart solutions, which has led to an evolution towards IoMT (Internet of Multimedia Things) [4]. In this field, the requirements of IoMT devices require low-cost solutions with restrictions in processing capacity and energy consumption, and with wireless connections to the network [5]. Moreover, the use of cloud computing applications is not feasible in image-processing applications due to latency (although 5G may change this) and privacy issues. This is highlighted in [6], where the importance of near-sensor computing is emphasized, relating IoMT to fog computing. The cloud cannot support and analyse the constant increase in data. Edge computing processes the data very close to the device, and sends only the significant information to higher levels. ETSI (European Telecommunications Standards Institute) has defined several examples of the use of mobile edge computing, one of which is video analysis. Examples of use are wearable cognitive assistance, behavioral analytics and telemedicine. In [6] a proof of concept of software/hardware co-design is proposed, using a Cortex A9, although the processing performed in the node is only image compression. In [7], IoT architectures are analysed, specifically for the development of Smart surveillance. In this paper, the use of small sensor nodes with limited computational capacity and power is highlighted, although only the aspects of media security and privacy in wireless sensor networks are examined. In [8] the authors use the concept of near sensors for machine-learning applications, arguing that a wide range of emerging edge intelligence applications is a necessary evolution towards replacing cloud computing applications. However, some of these applications generate very large amounts of data, as is the case with autonomous cars, which can reach 1 Gbps. Another limitation encountered with cloud computing is the use of private data, such as in biomedical devices or wearable cameras. In this last device, this capacity is also significant as it cannot guarantee 100% connection and, in addition, low energy consumption is extremely important. Cloud migration to the edge makes it possible to do the following:

- Reduce the vulnerability of data;
- Offer the possibility of customisation through specialisation of the hardware to reduce latency and energy consumption;
- Gain a huge reduction in bandwidth, avoiding the transmission of irrelevant information.

In this work, where trends to develop edge intelligence are analyzed, an Odroid XU4 with Linux and 2 GB of RAM is used. In [9] the authors highlight how embedded platforms are transforming and evolving quickly from standalone computer systems to become part of a smarter, more connected IoT that can be adopted and deployed in different environments, and are being adapted according to their restrictions and needs. A similar concept is that of the IoT-based multimedia applications (IoTMM), where connected industry is one of the 5 categories of application classified in [10], and where the importance of energy saving in the nodes (of limited resources) used is also highlighted. Another concept that is gaining in importance is that of embedded vision [11], which highlights the importance of SoC systems (system on chip) based on ARM architectures to revolutionize image and machine vision. There are therefore several areas where embedded systems, which are connected to the network, are required but which are characterized by restrictions in their computational capacity, consumption, and cost.

SoCs have evolved significantly in recent years, greatly influenced by the exponential increase in the smartphone market, to a point where today we have central processing units (CPUs) capable of running complete operating systems with their own graphical environment. These architectures have evolved from the original homogeneous architectures, to the current heterogeneous architectures, where several cores with different capacities and different energy requirements are mounted on the same chip. This property makes it the perfect choice to integrate IoMT systems into the Industry 4.0 paradigm, as it allows for enormous flexibility. The use of multicore systems also allows for improved energy efficiency based on a reduction in frequency achievable by spreading the work over several cores [12,13]. In this context, where flexibility is an important requirement, systems must be able to be reconfigured in a simple way, so that they can be adapted to applications with different computing requirements in an energy-efficient way.

**Figure 1:**
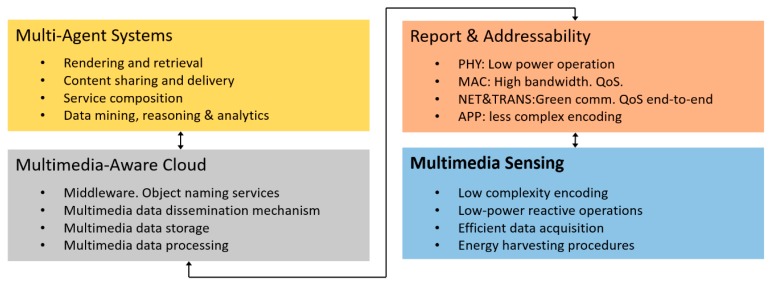
Levels on an Internet of Multimedia Things (IoMT) arquitecture [5].

Figure 1 shows an IoMT architecture divided into 4 levels. The lower level, multimedia sensing, is where this work is located. IoMT devices are resource constrained, low-cost, low-power and heterogeneous. They are limited in terms of power resources. However, they should be embedded with application- and context-aware intelligence, so that the multimedia content of the physical world is only acquired when necessary, minimizing the acquisition of redundant information. In the architecture proposed in [5] the only pre-transmission procedures considered are related to the compression of the captured multimedia information. In this work, IoMT technology is used to move from a cloud system to an edge system (or fog), using the processing capacity of the sensors to transmit only the information of interest, and only when that information exists. To develop this function, a method is proposed to configure the frequency governor of the cores, so that it satisfies the time requirements with the least possible energy consumption.

The predominant architecture in this type of ARM processor-based system is the *big.LITTLE* ARM architecture, where processes with less computational requirements are executed in the *LITTLE* cores. However, when increased computational capacity is needed, the process is executed in the *big* cores. Initially only the processor could internally decide on which core a process was running. In kernel switch scheduling (IKS), each pair of *big.LITTLE* cores was seen as a single virtual processor associated with a process, switching internally from one to the other according to these needs. More recently, global task scheduling (GTS) has become available [14], where each core, *big* or *LITTLE*, can execute tasks simultaneously. In [15] the differences in the IKS and GTS planning algorithms can be seen, as well as the different energy management techniques in mobile processing units. In these systems Dynamic voltage and frequency scaling (DVFS) is available, which is controlled by the governor used. The use of DVFS techniques has already been analyzed, but mainly in the area of smartphones, with a very specific workload and characteristics. In [16] the workload of smartphones and the influence of governors on heterogeneous multicore systems is analyzed, highlighting the over-design from a computational point of view in relation to the needs of smartphones. In [17], energy management methods are analysed from the point of view of the response time perceived by the user. Other works have attempted to characterize the user, such as [18] where machine-learning methods are used to identify and classify the user, and thus optimize energy consumption, or in [19] where a model for predicting satisfaction based on user history is presented. Other work on embedded and mobile systems is presented in [20], based on counter propagation networks to classify tasks and predict the best frequency for the system, or in the IoT environment in [21], based on extreme machine learning with the same objective. However, these proposals are not compared with the most suitable governors, when indicated, nor are their default parameters changed. Moreover, they select a fixed and static frequency for a given task, wasting the dynamic capacity to adjust this value when the workload is periodic but not symmetric. Thus in [20], *Ondemand* is used but not interactive, and it is not specified which parameters have been used. In [21], there is no specific information it has been compared to, but its results are compared to the governor that assigns the highest frequency, the performance governor, and therefore where any improvement in frequency scaling brings advantages.

In Ref. [15] there is another review of references using DVFS-based methods in applications ranging from 3D games on smartphones to wearable devices.

This paper does not propose a new governor that would be suitable for a multi-load and multi-application environment and that would require neural network training stages to assign statically the best working frequency for a given symmetric load. Instead, it aims to demonstrate how energy efficiency can be increased in devices by running IoMT applications, which have an asymmetric load by the very nature of the data they handle, using standard dynamic controllers, but setting the parameters of these controllers to achieve this efficiency. The paper presents a methodology to choose these parameters according to the load of the task, and its periodicity. Furthermore, instead of comparing only the energy improvements achieved, the results are explained through an analysis of the frequency histogram obtained with each method.

Section 2 reviews the architecture of ARM-based SoCs, and in particular the part corresponding to energy saving, defining a methodology to determine the parameters. The following section presents the experimental results obtained for different types of video sequences. Finally, the conclusions and future work to be done are presented.

## 2. Methodology for the Parameterization of the Governors

### 2.1. Architecture

**Figure 2:**
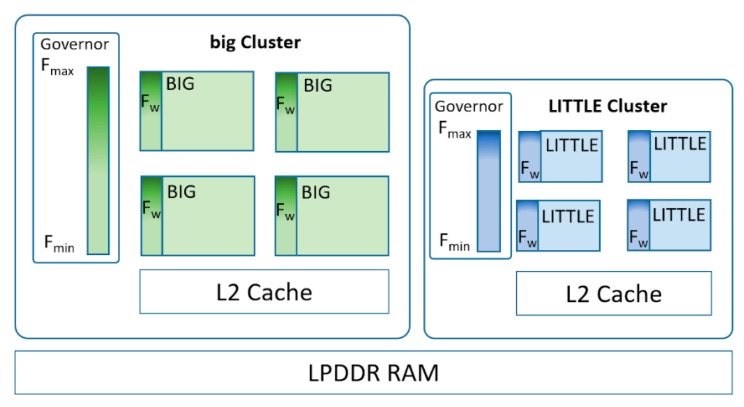
Typical big.LITTLE architecture, where governor regulates the frequency of each cluster.

In a big.LITTLE ARM architecture (see Figure 2) each type of core has a minimum frequency (*F_min_*), a maximum frequency (F_max_), and a range of frequencies available between these two, so that, depending on the policy applied by the governor, and the computer load required by the process, one particular frequency will be chosen as the working frequency (*f_w_*), a value that can change continuously during the execution of the task. These governors can apply a static or dynamic frequency assignment policy. As a static, there is the *Powersave* governor, where *f_w_* = *F_min_*, and the *Performance* governor, where *f_w_* = *F_max_*. The dynamic governors will make the f_w_ value go up and down through the available frequencies between *F_min_* and *F_max_* with the objective of executing the tasks in a satisfactory time alongside a reduction in the energy consumption. Common examples of dynamic policies are the *Ondemand*, *Conservative*, and *Interactive* governors. These are naive algorithms [22], which means that appropriate configuration may give a significant improvement in consumption. Using *Ondemand*, if the load on the core exceeds a certain threshold, *f_w_* = *F_max_* will be set, and a gradual reduction of *f_w_* will be performed until *f_w_* = *F_min_* is reached, as the load on the core decreases. *Conservative* has a more gradual way of raising the *f_w_* value, and a progressive decrease when the load drops from the lower threshold. The *Interactive* governor was designed for interactive workloads that require a fast reaction in response to user actions. In addition, the procedure for adjusting the *f_w_* value is in the kernel with the highest priority, in order to avoid delays in response. This governor, has a series of parameters that allow the operation to be regulated according to the relationship between performance and energy to be achieved. Table 1 shows a description of these parameters, and Table 2 shows the default values used and those proposed here.

In Algorithm 1 there is a description of the operation of the algorithm which aims to determine the value of *f_w_* so that it meets the requirements of applications while reducing consumption. Each load-sampling interval (*TR*) is checked to see if this load is above the *GHL* threshold. If it is, the frequency is raised to the value of *F_hs_*. Once this frequency has been established, the system waits for a time determined by *AHD*, to re-evaluate the use, and if it continues to exceed the threshold, it increases the *f_w_* value again, which is already already above *F_hs_*. value, and may reach *F_max_*. If the threshold is not exceeded, the system waits for *MST*, and if the load remains below the threshold, the value of *f_w_* will be reduced. Usually this governor is configured so that *F_hs_* = *F_max_*, as also indicated in [15], which improves the system’s reaction time, but does not allow the exploitation of the range of intermediate frequencies between the maximum and minimum, providing a very fast response, but also with the highest power consumption.
**Algorithm 1** Interactive governor algorithm1: Set core working frequency (*f_w_*) between *F_min_* and *F_max_*2: for every *TR*
**do**3:  U ← current_CPU_Utilization4:  **if** (U > *GHL*) **then**5:   *f_w_ = F_hs_*6:   wait *AHD*7:   U ← current_CPU_Utilization8:   **if** (U > *GHL*) **then**     increase *f_w_*9:   **end if**10:  **else**11:   wait (*MST*)12:   **if** (U < *GHL*) **then**
     decrease *f_w_*13:   **end if**14:  **end if**15: **end for**

### 2.2. Methodology for Parameter Selection

**Figure 3:**
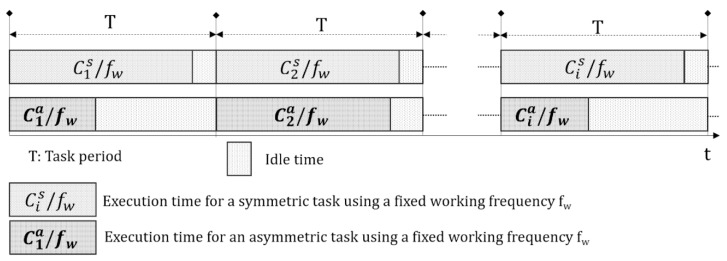
Types of IoMT system loads.

The approach is that the flexible node IoMT will be executing a certain task at a given time with a periodicity T. The algorithm to be executed in each period may be symmetric and invariant to the content, so that its execution time ($C_{i}^{s}$) in each period T will be constant if used at the same frequency f_w_ ($C_{i}^{s}\approx C^{s},\;\forall i\;/\;f_{w}^{i}=f_{w}\forall i$). In this case, a static governor can be chosen using a fixed frequency *f_w_*, between *F_min_* and *F_max_* so that ${C^{s}}_{\sim }^{<}T$, and in this way the temporal requirements of the application are satisfied. Given the quadratic relationship between energy and frequency, the lower the *f_w_*, the lower the consumption. In the case of an asymmetric algorithm, where the execution time ($C_{i}^{a}$) is not constant but depends on the content of the images, the use of another certain fixed frequency *f_w_* means that there is sometimes too much idle time, while in other cases there may be very little idle time or the time T may even be exceeded. Figure 3 shows these two situations graphically.

The process for determining the value of *f_w_* when the load is symmetrical is described in [23] in which recommendations for asymmetrical loads are also given. In the case of the asymmetrical load $C_{i}^{a}$, this is defined by the average ${\hat{C}}^{a}$, the standard deviation $\sigma_{C^{a}}$, and the worst case $C_{wc}^{a}$ ($C_{wc}^{a}=\max\left(C_{i}^{a}\right)$).

The proposed relationship to choose the value of the working frequency is [23]:

(1) $$f_{w}=F_{i\;}:C_{wc}^{a}<T$$

That is, the frequency *F_i_* is chosen which allows that even in the worst case, the execution time is less than the period T of the tasks. However, to satisfy the worst case, in a static governor, a high value, close to *F_max_*, may have to be taken and thus the idle time is considerable, which will mean a significant waste of energy. In the case of using a dynamic governor, the parameters *F_hs_* and *AHD* will be modified in order to satisfy $C_{wc}^{a}<T$, at the same time achieving an important energy saving. The first proposal for the value of *F_hs_* in this paper is:

(2) $${F^{\prime }}_{hs}=F_{i\;}:{\hat{C}}^{a}<T$$

That is to say, the frequency *F_i_* is chosen, which guarantees that the average time is less than the period. Although a value close to the average is used, this is for a determined value of *f_w_*. Choosing this value for *F_hs_*, if the computational load for processing an image is higher, the value of *f_w_* will change from *F_hs_* to higher values, meaning that the execution time will be reduced in comparison with the time that would be obtained with a static governor using *f_w_* = *F′_hs_*. In whichever case, with this selection, the deadline will not be met on occasions. Another possible relationship to reduce the possibility of the deadline not being met is:

(3) $${F^{\prime \prime }}_{hs}=F_{i\;}:{\hat{C}}^{a}+3\sigma_{C^{a}},\;<T$$

In this case a value of *F_hs_* higher than with Equation (2) will be chosen, reducing the chances of missing the deadline at the cost of higher energy consumption. The other important parameter is the value for the time that this frequency will remain in use, before checking that the use is still high and therefore raising the frequency again.

(4) $$AHD=A^{\prime }\approx T$$

In this way, we can be sure that we are working for a greater period of time with the value of *F_hs_*, before increasing the value of *f_w_* towards *F_max_*.

## 3. Experimental Results

### 3.1. Equipment and Sequences

An Odroid XU4 was used for the experiments, the main features of which can be seen in Table 3.

**Figure 4:**
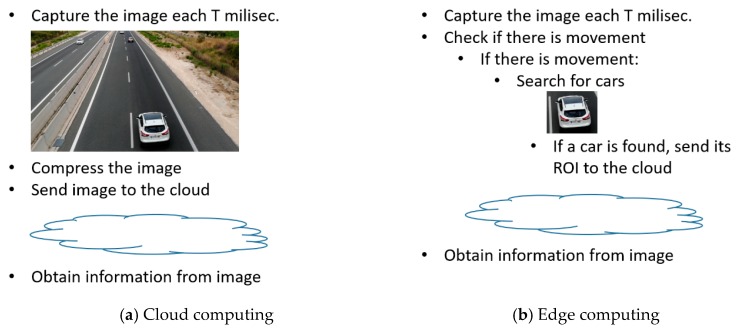
Examples of different behavior on cloud and edge computing.

The haartraining algorithm using OpenCV was used as a load on a video sequence. The video sequence is from a highway, where the passage of vehicles is controlled. In a cloud system, the system would capture and send all the images to the cloud to be correctly processed there. Images like that shown in Figure 4a would involve the entire image being sent to the central office for processing. In the fog computing system, the algorithm looks for the cars in the sequence, and only sends the ROIs (regions of interest) of the images where it has located vehicles, as can be seen in Figure 4b. Since it is a process that is costly in terms of computing requirements, this vehicle search process has not been executed on all the images, but only on those where a threshold of change between the images I_i_ and I_i+1_ is exceeded (it is assumed that there is no alternative sensorization that indicates the presence of vehicles, either because it is a provisional installation where an attempt is made to economize on the installation, or because of the difficulties that may exist in the location of presence sensors that can perform this function. Therefore, this function will be carried out by means of multimedia processing [24]). 

This same sequence was then used, but processed in three different ways, and henceforth in this work will be described as three different videos. In the first (vid1) an artificially high change threshold has been used, so it does not manage to detect vehicles and is, therefore, equivalent to a video sequence with very low activity. In the second (vid2), the threshold is set to an appropriate value, so all the images where there are changes are processed and movement of cars is detected, and the ROI of the vehicles found is also sent by WiFi. In this sequence, moments of higher activity are alternated with moments of less activity. The third (vid3) is a part of the same sequence with high activity, which is repeated several times and is thus considered to be a scene with high activity.

Table 4 shows the energy consumption in Wh made in one hour of video transmission, calculated by extrapolating the data from the duration of the video to a one-hour video, and this will also be the way energy consumption is expressed in the rest of the cases, so that the comparison is easier to perceive. Only the consumption in the IoMT device is considered here, and not the consumption generated from having to process in the cloud servers all the images in search of vehicles, a task that will not be necessary in the fog computing approach. 

Table 5 shows the values obtained with the Performance governor using different values of *F_max_* for the sequence Vid3. As can be seen, the value of ${F^{\prime }}_{hs}\;\mathrm{would}\;\mathrm{be}\;1.0\;\mathrm{GHz}$, while the value of ${F^{\prime \prime }}_{hs}\;\mathrm{would}\;\mathrm{be}\;1.6\;\mathrm{GHz}.$ A value of *T* = 125 ms has been used in the sequence, so the value of *AHD* chosen is 120 ms.

### 3.2. Results

#### 3.2.1. Results Obtained for a Sequence of Medium Activity

In the sequence denominated Vid2, the values obtained by the *Performance* and *Ondemand* governors can be seen in Table 6.

Table 7 shows the results using the *Interactive* governor, with its default parameters (*F_hs_* = 2.0 GHz), and the values obtained for lower values of *F_hs_* while maintaining the rest of the default parameters. As can be seen in the table, the energy cost is higher than that obtained with the OnDemand governor, very close to that of the Performance governor, with similar temporal results. In the case of choosing a value of *F_hs_* in this configuration which would provide a power consumption similar to that of OnDemand, as with *F_hs_* = 800 MHz, the values of ${\hat{C}}^{a}$ and $\sigma_{C^{a}}$ are significantly worse.

Table 8 shows the results using the interactive governor, with the parameters proposed in the previous section (*F′_hs_* = 1.6 GHz or *F″hs* = 1.0 GHz, where *AHD* = 120 ms. and the rest of parameters as conf2), and the values obtained for other *F_hs_* values. As can be seen, using the proposed parameters compared to the default ones, the power consumption using *F′_hs_* is reduced by 41% compared to *Performance* and 8% compared to *Ondemand*, while the temporal values also show better performance compared to the *Ondemand* governor. Using *F″_hs_* it consumption is reduced by 50% compared to *Performance* and 15% compared to *Ondemand*, maintaining a temporal behavior similar to *Ondemand*. The same values of *F′_hs_* give better energy consumption and behaviour of conf2 compared to conf1.

**Figure 5:**
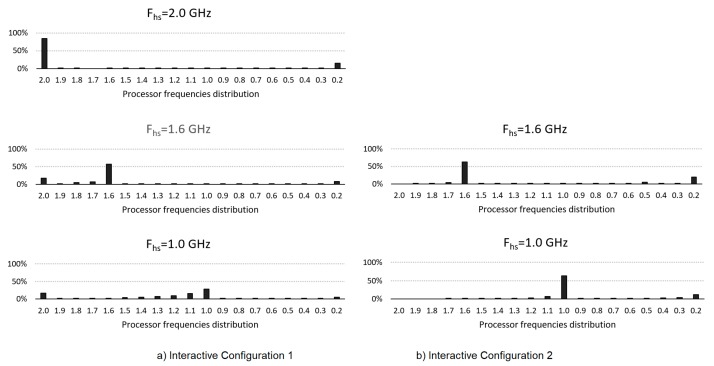
Core frequencies distribution for each configuration for Vid2.

Figure 5 shows the distribution histograms of the core frequencies used with the two configurations: (a) with *F_hs_* = 2 GHz, (b) with *F′_hs_* = 1.6 GHz and *F″_hs_* = 800 MHz. The same frequencies are included with configuration 1 for comparison purposes. As can be seen, when using *F_hs_* = *F_max_* in configuration 1 (and as it is a process of high computational requirements) the frequency remains at its maximum value most of the time, being at its *F_min_* value, a very small part of the time. Using configuration 2 and the values *F′_hs_* and *F″_hs_*, it is clear that most of the time the core will work with that frequency, although sometimes it is necessary to increase the frequency value, but without reaching *F_max_* at any time. Using the same values of *F′_hs_* and *F″_hs_* but with configuration 1 (default), the results show how the core, even when remaining for some time in that *F_hs_* frequency, the lower value of *AHD* means that it passes more easily to higher frequencies, including *F_max_*, so the consumption is higher but without providing a significant time difference. The reduction of the *MST* value can also be seen in lower use between *F_hs_* and *F_min_* increasing the time in *F_min_*, which also favours the energy reduction achieved with configuration 2.

**Figure 6:**
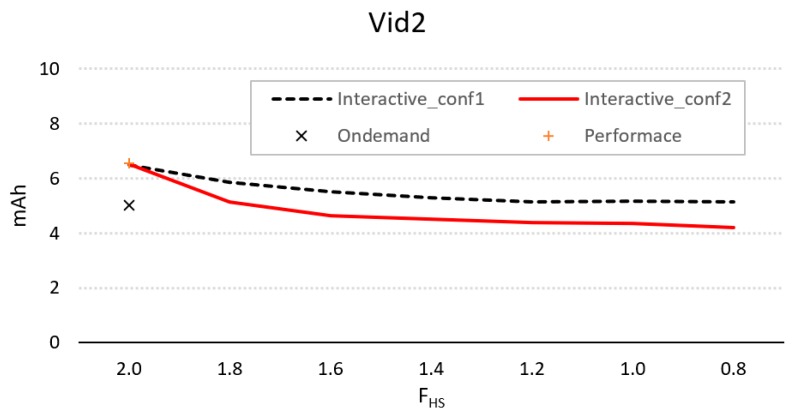
Evolution of energy consumption for the interactive controller for configurations 1 and 2, using different values of *F_hs_*, compared to consumption using *Ondemand* and *Performance*.

Figure 6 shows the difference in energy consumption for the parameters of conf1 and conf2, using different values of *F_hs_* and compared to *OnDemand* and *Performance*. As can be seen, only conf2 provides better energy performance compared to *Ondemand*, and this is achieved by using an *F_hs_* < *F_max_*.

#### 3.2.2. Results Obtained for a Low-Activity Sequence

In the Vid1 sequence, when there is no activity in the whole video sequence, the values obtained by the *Performance* and *Ondemand* controllers are shown in Table 9. As can be seen, the values are lower than those obtained for the cloud computing solution, since only the images are captured and the existence of activity is verified, but if there is no activity, neither transmission of images or ROIs is produced, meaning that the energy consumption is lower.

Table 10 shows the results using the Interactive governor, with its default parameters (*F_hs_* = 2.0 GHz), and the values obtained for lower values of *F_hs_*, while maintain the rest of the default parameters. As can be seen in the table, the same results are obtained as in the previous case. The energy cost is higher than that obtained with the *OnDemand* governor, and very close to that of the *Performance* governor, with quite similar temporal results. In the case of using an *F_hs_* that provides similar energy consumption, as is the case of 1.6 GHz, better temporal behavior can be appreciated.

Table 11 shows the results using the *interactive* governor, with the parameters proposed in the previous section (*F′_hs_* = 1.6 GHz o *F″_hs_* = 1.0 GHz, where *AHD* = 120 ms. and the rest of the parameters as in conf2), and the values obtained for other *F_hs_* values. As can be seen, using the proposed parameters compared to the default parameters, the energy consumption using *F′_hs_* is reduced by 62% compared to the *Performance* and 18% compared to the *Ondemand*. Although the average time is 33% worse than *Ondemand*, it is in no danger of failing to meet the deadline. Using *F″_hs_* energy consumption is reduced by 43% compared to *Performance* and 5% compared to *Ondemand*, maintaining a temporal behavior somewhat better than *Ondemand*. In any case, the same values of *F_hs_* give better consumption and behavior of conf2 compared to conf1.

**Figure 7:**
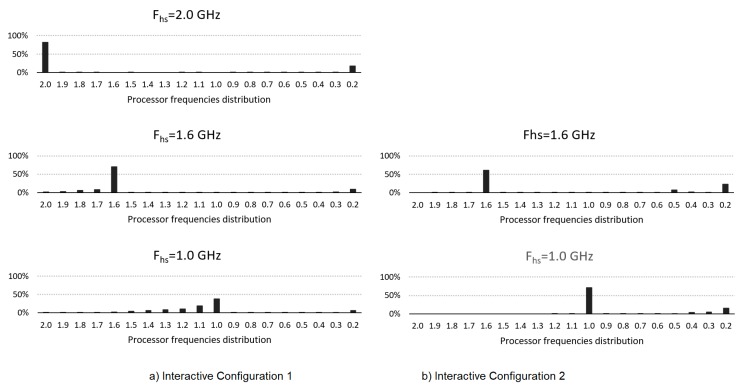
Core frequency distribution for each configuration for Vid1.

Figure 7 also shows the frequency distribution histograms as above. As can be seen, using conf2 the highest frequency is practically *F_hs_*, while with conf1, this value is exceeded as there is also a high use in a shorter evaluation time, as is the case with conf1.

**Figure 8:**
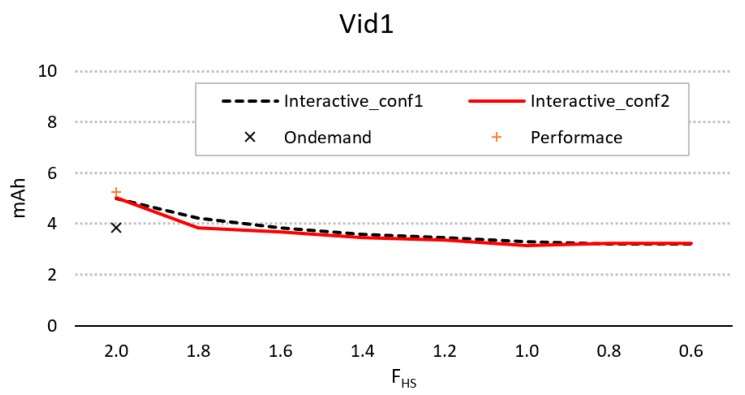
Evolution of energy consumption for the interactive controller for configurations 1 and 2, using different values of *F_hs_*, compared to consumption using *Ondemand* and *Performance*.

Figure 8 shows the difference in energy consumption for the parameters of conf1 and conf2, using different values of *F_hs_* and compared to *OnDemand* and *Performance*. As can be seen, with no activity in the sequence, both conf1 and conf2 provide better energy performance compared to *Ondemand*, although in both cases it is also necessary that *F_hs_* < *F_max_*.

#### 3.2.3. Results Obtained for a Sequence with High Activity

In what is considered sequence 3, when there is a lot of activity throughout the video sequence, the values obtained by the *Performance* and *Ondemand* controllers can be seen in Table 12.

Table 13 shows the results using the *Interactive* governor, with its default parameters (*F_hs_* = 2.0 GHz), and the values obtained for lower values of *F_hs_* maintaining the rest of the default parameters. As can be seen in the table, the same results are obtained as in the previous case. The energy cost is higher than that obtained with the *OnDemand* governor, very close to that of the Performance governor, with quite similar temporal results.

Table 14 shows the results using the *Interactive* governor, with the parameters proposed in the previous section (*F′_hs_* = 1.6 GHz o *F″_hs_* = 1.0 GHz, with *AHD* = 120 ms. and the rest of parameters as conf2), and the values obtained for other *F_hs_* values. As can be seen, using the proposed parameters compared to the default parameters, the energy consumption using *F′_hs_* is reduced by 26% compared to the *Performance* and 22% compared to the *Ondemand*. The average time is 21% worse, and furthermore, as expected, not all deadlines are met, although these are not fully met using either *Ondemand* or *Performance*. Using *F″_hs_* energy consumption is reduced by 22% compared to *Performance* and by 12% compared to *Ondemand*, maintaining a slightly better time performance than *Ondemand* and meeting all deadlines. In this case, the same values of *F_hs_* give a better consumption with configuration 2, while the temporal behavior is very similar.

**Figure 9:**
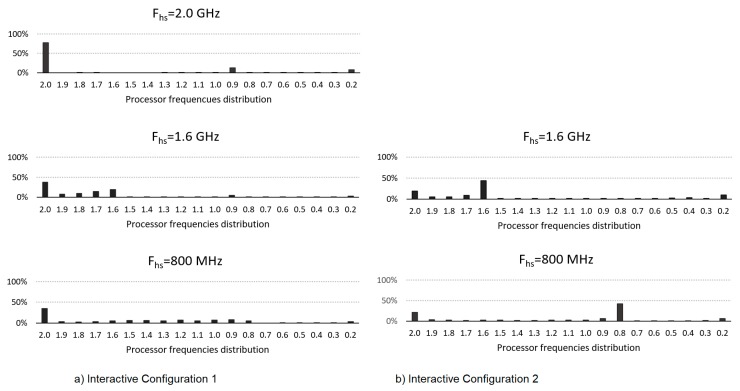
Core frequencies distribution for each configuration for Vid3.

Figure 9 also shows the frequency distribution histograms as above. As can be seen, as it requires a greater computational load, in both configurations *F_max_* is reached, although the percentage of use is higher in conf1 compared to conf2, where *F_hs_* is still the most used frequency, although now this frequency is at times exceeded. 

**Figure 10:**
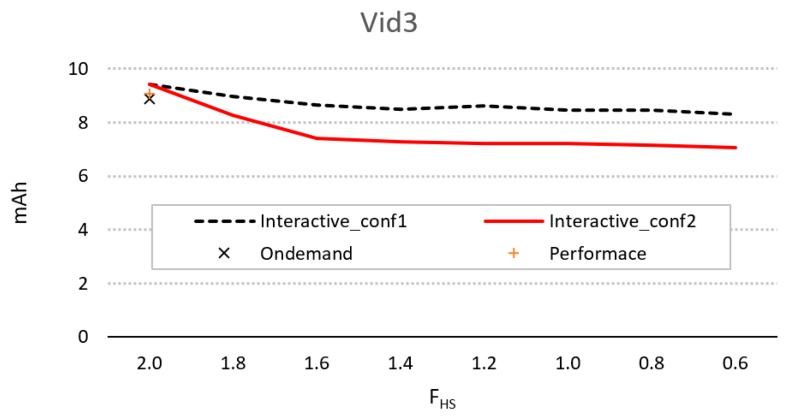
Evolution of energy consumption for the interactive governor for configurations 1 and 2, using different values of *F_hs_*, compared to consumption using *Ondemand* and *Performance*.

Figure 10 shows the difference in energy consumption for the parameters of conf1 and conf2, using different values of *F_hs_* and compared to *OnDemand* and *Performance*. The figure shows that as there is a high activity in the sequence, the consumption using *Ondemand* is close to that of *Performance*. With this workload, both conf1 and conf2 can provide better energy performance compared to *Ondemand*, although in both cases it is also necessary that *F_hs_* < *F_max_*. 

## 4. Conclusions and Future Work

The use of smart sensors in the field of industry, in particular in near sensor computing or fog computing, has great potential for use in the future. The requirements determined by industry 4.0 require the flexibility of the sensors so that they can be used for different types of tasks. Multicore architectures, whether *big.LITTLE* or not, allow the field of use of these devices to be extended. Thus, with respect to a cloud computing solution using *Performance*, savings of 178% are achieved for vid1, 101% for vid2, and 21% for vid3. Thus, compared to a cloud computing solution, a high energy saving is achieved, a key aspect in this field, although it depends on the system load. As for the solution based on edge computing, the default configurations are designed for smartphones. The use of the *Interactive* governor allows different computing loads to be dealt with efficiently. If the parameters of this governor are also set taking into account the load with which they have to work at a given time in an application in the 4.0 industry, it is possible to significantly improve energy savings while maintaining or even improving the temporal response of the system. This is demonstrated by the differences between configuration 2 used with the interactive governor, and configuration 1 with this same governor, or using *Performance* or *Ondemand*. Comparing the proposed *Interactive* configuration with respect to *Performance*, which is the one used by default by Exynos_cpufreq (see Table 3), savings are achieved from 62% for low-activity sequences to 26% for very high-activity sequences. Comparing the proposed *Interactive* configuration (conf2) with the default *Interactive* configuration (conf1), savings are achieved from 58% for low-activity sequences, to 31% for very high-activity sequences. 

This paper presents a method to determine the parameters of the *Interactive* governor, so that the temporal requirements of the applications are maintained, improving significantly the energy consumption. This improvement makes an edge computing solution even more efficient than cloud computing. The use of these SoCs in industry 4.0 allows a high degree of flexibility; they could be used, depending on the moment, using only a *LITTLE* core in applications with very low computing requirements, to other applications, such as that shown in Section 3, where the 4 big cores are used, which represents a very high range of applications. 

The parameter selection method shown is based on the experimental information obtained with a video sequence. As a future work, and with the aim of achieving a flexible and more autonomous and reconfigurable system, the authors aim to develop a workload analysis system so that it is possible to select not only the parameters of the governor, but also to choose which type of cores and how many cores to use to achieve the desired performance, thus increasing energy savings.

## Figures and Tables

**Table 1:** Interactive parameters.

Parameter	Symbol	Description
Hispeed_freq	*F_hs_*	Value of *f_w_* initially chosen as soon as the core load exceeds a certain load value
go_higspeed_load	*GHL*	Load threshold to increase frequency
above_highspeed delay	*AHD*	Time during which, if the load continues to exceed the threshold, the frequency *f_w_* will be raised again until *F_max_* is reached
timer_rate	*TR*	Load sampling interval if the core is not idle
min_sample_time	*MST*	Minimum time at a certain frequency before reducing its value

**Table 2:** Interactive default parameters values and proposed values.

Parameter	Symbol	Default Value Configuration 1 (conf1)	Proposed Value Configuration 2 (conf2)
Hispeed_freq	*F_hs_*	*F_max_*	F′
go_higspeed_load	*GHL*	99%	99%
above_highspeed delay	*AHD*	20 ms	A′ ms
timer_rate	*TR*	20 ms	10 ms
min_sample_time	*MST*	80 ms	10 ms

**Table 3:** Big.LITTLE Cores of Odroid XU4.

Processor Type	Properties
Big Core	4 Cortex A15 (ARMv7 ISA) up to 2.0 GHz
	CPU Varian 0x2. CPU Part 0xC0F
	Frequencies from 0.2 GHz to 2.0 GHz steps: 100 MHz
Little Core	4 Cortex A7 (ARMv7 ISA) up to 1.4 GHz
	CPU Varian 0x0. CPU Part 0xC07
	Frequencies from 0.2 GHz to 1.4 GHz steps: 100 MHz
Both	Implementer 7. Revision 3
	Scaling driver: Exynos_cpufreq

**Table 4:** Energy used through cloud computing solution.

Ondemand	Performance
Vid2	
7.78	8.75

**Table 5:** Values obtained for performance governor with different *F_max_* values. Vid3.

*F_max_*(GHz)	2.0	1.8	1.6	1.4	1.2	1.0	0.8	0.6
Energy	9.035	7.732	6.345	5.555	5.243	4.755	5.119	5.510
${\hat{C}}^{a}$	63.70	51.90	58.97	66.83	83.77	**102.38**	125.88	166.80
$\sigma_{C^{a}}$	32.91	13.91	19.56	23.88	37.53	54.675	70.95	91.70
$C_{max}^{a}$	265.75	119.54	183.87	283.80	301.72	467.15	685.14	755.19
$C_{min}^{a}$	36.58	40.19	45.17	49.09	55.78	65.86	80.91	107.04
${\hat{C}}^{a}+3\sigma_{C^{a}}$	162.45	93.64	**117.66**	138.47	196.37	266.40	338.75	441.89

**Table 6:** Performance and OnDemand governors with *F_max_*. Vid2.

	Energy	${\hat{C}}^{a}$	$\sigma_{C^{a}}$	$C_{max}^{a}$	$C_{min}^{a}$
Performance	6.564	22.45	5.84	62.00	19.92
OnDemand	5.015	42.70	20.84	117.81	21.14

**Table 7:** Interactive governor with configuration 1 and different *F_hs_* values. Vid2.

*F_hs_*(GHz)	2.0	1.8	1.6	1.4	1.2	1.0	0.8	0.6
Energy	6.482	5.851	5.510	5.288	5.152	5.169	5.152	
${\hat{C}}^{a}$	43.90	48.64	39.53	42.35	44.97	47.15	59.64	52.96
$\sigma_{C^{a}}$	9.16	8.77	9.38	9.91	11.15	11.47	12.38	15.31
$C_{max}^{a}$	99.24	58.15	60.34	61.20	152.63	70.76	73.02	113.82
$C_{min}^{a}$	19.92	19.87	19.91	19.82	19.95	20.05	19.88	20.03

**Table 8:** Interactive governor with configuration 2 and different *F_hs_* values. Vid2.

*F_hs_*(GHz)	2.0	1.8	1.6	1.4	1.2	1.0	0.8	0.6
Energy	6.533	5.135	4.640	4.520	4.384	4.350	4.213	4.112
${\hat{C}}^{a}$	33.31	39.78	37.00	40.50	45.18	43.19	63.36	81,51
$\sigma_{C^{a}}$	4.12	4.00	3.57	3.86	5.38	7.22	7.17	9.07
$C_{max}^{a}$	52.84	65.50	63.47	60.49	86.27	106.29	96.37	125.35
$C_{min}^{a}$	20.23	21.08	21.71	25.34	22.07	27.21	25.89	23.56

**Table 9:** Performance and OnDemand governors with F_max_. Vid1.

	Energy	${\hat{C}}^{a}$	$\sigma_{C^{a}}$	$C_{max}^{a}$	$C_{min}^{a}$
Performance	5.25	21.03	1.05	61.63	20.29
OnDemand	3.85	39.08	18.63	140.99	20.02

**Table 10:** Interactive governor with conf1 and different *F_hs_* values. Vid1.

*F_hs_*(GHz)	2.0	1.8	1.6	1.4	1.2	1.0	0.8	0.6
Energy	4.98	4.21	3.83	3.58	3.44	3.30	3.20	
${\hat{C}}^{a}$	38.18	36.32	37.15	39.71	40.42	41.75	43.17	47.26
$\sigma_{C^{a}}$	7.02	8.45	8.01	8.40	8.90	9.65	10.17	12.88
$C_{max}^{a}$	98.93	101.41	59.36	91.12	132.54	117.12	143.39	108.28
$C_{min}^{a}$	20.93	21.00	21.42	22.72	24.08	24.04	24.16	23.78

**Table 11:** Interactive governor with conf2 and different *F_hs_* values. Vid1.

*F_hs_* (GHz)	2.0	1.8	1.6	1.4	1.2	1.0	0.8	0.6
Energy	5.03	3.83	3.66	3.46	3.36	3.14	3.24	3.24
${\hat{C}}^{a}$	30.43	33.44	35.09	37.73	40.82	44.63	51.99	63.93
$\sigma_{C^{a}}$	3.32	2.66	2.39	2.64	3.08	2.98	3.12	4.48
$C_{max}^{a}$	59.19	56.23	49.01	62.30	53.28	92.88	94.39	94.65
$C_{min}^{a}$	20.82	22.67	24.87	27.75	31.41	37.07	42.21	47.87

**Table 12:** Performance and OnDemand governors. Vid3.

	Energy	${\hat{C}}^{a}$	$\sigma_{C^{a}}$	$C_{max}^{a}$	$C_{min}^{a}$
Performance	9.065	63.70	32.91	265.75	36.57
On Demand	8.888	71.77	24.10	219.22	38.44

**Table 13:** Interactive governor with configuration 1 and different *F_hs_* values. Vid3.

*F_hs_* (GHz)	2.0	1.8	1.6	1.4	1.2	1.0	0.8	0.6
Energy	9.421	8.976	8.665	8.488	8.621	8.443	8.433	8.312
${\hat{C}}^{a}$	60.24	56.44	56.48	59.30	62.05	63.36	65.57	68.53
$\sigma_{C^{a}}$	18.51	14.96	13.36	15.41	16.31	16.83	17.44	20.88
$C_{max}^{a}$	199.93	147.56	126.27	152.31	148.18	169.17	180.76	195.27
$C_{min}^{a}$	38.57	38.57	38.95	38.62	38.72	38.68	39.07	38.19

**Table 14:** Interactive governor with configuration 2 and different *F_hs_* values. Vid3.

*F_hs_* (GHz)	2.0	1.8	1.6	1.4	1.2	1.0	0.8	0.6
Energy	9.421	8.265	7.421	7.288	7.200	7.183	7.154	7.056
${\hat{C}}^{a}$	57.17	54.89	57.15	61.36	65.67	73.41	81.40	94.58
$\sigma_{C^{a}}$	17.00	10.02	10.60	11.62	12.24	19.94	21.96	30.58
$C_{max}^{a}$	146.78	106.44	128.28	145.74	145.74	259.37	202.48	332.00
$C_{min}^{a}$	38.62	40.60	40.32	39.67	40.11	40.00	39.40	39.54
